# Linear viscoelasticity - bone volume fraction relationships of bovine trabecular bone

**DOI:** 10.1007/s10237-016-0787-0

**Published:** 2016-04-18

**Authors:** Krishnagoud Manda, Shuqiao Xie, Robert J. Wallace, Francesc Levrero-Florencio, Pankaj Pankaj

**Affiliations:** 1 School of Engineering, The University of Edinburgh, The King’s Buildings, Edinburgh, EH9 3DW UK; 2 Department of Orthopaedics, The University of Edinburgh, Chancellors building, Edinburgh, EH16 4SB UK

**Keywords:** Creep, Relaxation, Time-dependent, Linear viscoelastic, Bovine trabecular bone, BV/TV

## Abstract

Trabecular bone has been previously recognized as time-dependent (viscoelastic) material, but the relationships of its viscoelastic behaviour with bone volume fraction (BV/TV) have not been investigated so far. Therefore, the aim of the present study was to quantify the time-dependent viscoelastic behaviour of trabecular bone and relate it to BV/TV. Uniaxial compressive creep experiments were performed on cylindrical bovine trabecular bone samples ($\textit{n}\,{=}\,13$) at loads corresponding to physiological strain level of 2000 ${\upmu }{\upvarepsilon }$. We assumed that the bone behaves in a linear viscoelastic manner at this low strain level and the corresponding linear viscoelastic parameters were estimated by fitting a generalized Kelvin–Voigt rheological model to the experimental creep strain response. Strong and significant power law relationships ($r^2\,{=}\,0.73,\ p\,{<}\,0.001$) were found between time-dependent creep compliance function and BV/TV of the bone. These BV/TV-based material properties can be used in finite element models involving trabecular bone to predict time-dependent response. For users’ convenience, the creep compliance functions were also converted to relaxation functions by using numerical interconversion methods and similar power law relationships were reported between time-dependent relaxation modulus function and BV/TV.

## Introduction

The mechanical behaviour of bone is generally modelled using linear time-independent isotropic elasticity (Pankaj 2013). Cellular structure of trabecular bone has led to a number of studies which empirically relate Young’s modulus to density of the bone. Typically, in the development of subject-specific models computed tomography (CT) attenuations, which are known to relate to bone density, are used to assign inhomogeneous elastic properties (Taddei et al. 2007; Schileo et al. 2008; Tassani et al. 2011). In vitro validation experiments have shown that such assignment results in satisfactory prediction of response (Yosibash and Trabelsi 2012). However, it has been recognized that the mechanical response of bone when subjected to loads is not instantaneous; it is time-dependent (Schoenfeld et al. 1974; Zilch et al. 1980). The study of time-dependent elastic behaviour, also referred as viscoelastic behaviour, is of interest in several contexts such as: loosening of orthopaedic implants (Norman et al. 2006); compatibility of bone substitutes (Phillips et al. 2006); and energy absorption due to dynamic loads (Linde et al. 1989).

Time-dependent behaviour also plays a significant role in nontraumatic fractures or vertebral deformities (Pollintine et al. 2009; Luo et al. 2012) due to prolonged load over time with age and high-energy impact type fractures such as those due to a fall (Parkkari et al. 1999). A better understanding of the time-dependent behaviour of bone would help to further elucidate the mechanism of such fractures. Trabecular bone plays an important role in the stability of many implants, e.g. treatment of proximal femoral fractures (using sliding hip screws or gamma nails) (Jenkins et al. 2013; Goffin et al. 2013, 2014), and in total knee replacements (Meneghini et al. 2011). Fracture fixation that involves pins and screws traversing through the bone can result in large stresses at the implant–bone interface (Cheal et al. 1992; MacLeod et al. 2012) during surgery. These bone stresses, in a relatively short time period, may reduce due to the stress relaxation of the bone and jeopardize the initial fixation (Norman et al. 2006), which is based on interference fit. Further cyclic loading may affect the bone-implant interface mechanics as the bone and the implant do not have the same time-dependent response to loads, resulting in creep deformation and eventually implant loosening.

The viscoelastic response of trabecular bone has been experimentally measured using: relaxation tests (Schoenfeld et al. 1974; Zilch et al. 1980; Deligianni et al. 1994; Bredbenner and Davy 2006; Quaglini et al. 2009) in which time-dependent varying force due to applied constant strain is measured over time; creep tests (Bowman et al. 1994, 1998; Yamamoto et al. 2006) in which time-varying strain due to applied constant load is measured over time; and dynamic mechanical analysis (Guedes et al. 2006; Kim et al. 2012, 2013) in which the lag between sinusoidal stress and strain is measured over a frequency range. It has been observed that trabecular bone creeps even at smaller load levels corresponding to physiological activities (Yamamoto et al. 2006; Pollintine et al. 2009; Kim et al. 2012).

However, unlike time-independent elasticity, the link between time-dependent viscoelastic properties of trabecular bone with bone volume fraction (BV/TV) or density have not been previously reported. Such relationships will permit use of viscoelastic material models in the finite element (FE) analysis of bone and bone-implant systems where required. It is important to note that while most commercial FE packages are capable of simulating viscoelastic behaviour, this feature is not commonly used as the required parameters are not readily available. This study aims to fill this gap.

Therefore, the primary objectives of the present study are: (1) to quantify the viscoelastic properties of the trabecular bone from a series of uniaxial compressive creep experiments on bovine trabecular bone and to relate these to BV/TV; and (2) use interconversion methods to establish similar mathematical relations between BV/TV of the bone and relaxation functions.

## Materials and methods

### Creep experiments

Fresh proximal femora from bovine, female, under 30 months-old when killed, were obtained from a local abattoir and were stored at $-20 ^{\circ } \mathrm{C}$ until utilized. The bones were allowed to thaw to room temperature before the femoral heads and trochanters were removed using a hacksaw. Transmission radiographs were then taken of the whole femoral head. These images indicated the trabecular principal axis for specimen extraction. Care was taken to ensure that a central core was extracted along this axis using a diamond core drill bit (Starlite, Rosemont, IL, USA). Four additional cores were extracted parallel to this first central core from each of the two bovine femoral heads, and three cores from another two trochanters using the diamond core drill bit. Once extracted the cores were examined for the presence of a growth plate, and if found this was removed during sample preparation. A low speed rotating saw (Buehler, Germany) was used to create parallel sections. The cylindrical bone samples in total $n\,{=}\,13$ were of diameter 10.6$\,{\pm }\,$0.1 mm and mean height of 25.1$\,{\pm }\,$2.1 mm. The heads and trochanters were kept hydrated while drilling in a custom made holding clamp to mitigate temperature damage. Brass end-caps were glued to each end of the sample using bone cement (Simplex, Stryker, UK) to minimize end-artefacts during compression testing (Keaveny et al. 1997). Effective length (22.2$\,{\pm }\,$2.1 mm) of each specimen was calculated as the length of the sample between the end-caps plus half the length of the sample embedded within the end-caps (Keaveny et al. 1997), and this effective length was used in calculating average strains. A water bath filled with phosphate-buffered solution (PBS) was used around each sample to keep it hydrated during imaging and through all phases of mechanical testing.

Before mechanical testing high resolution microcomputed tomography ($\upmu$CT) scans were taken of each sample using a Skyscan 1172 $\upmu$CT scanner (Bruker microCT, Kontich, Belgium). The following scan parameters were used: voxel resolution 17.22 ${\upmu }$m, source voltage 100 kV, current 100 ${\upmu }$A, exposure 1771 ms with a 0.5 mm aluminium filter between the X-ray source and the specimen. Image quality was improved by using 2 frame averaging. The images were reconstructed with no further reduction in resolution using Skyscan proprietary software, nRecon V1.6.9.4 (Bruker microCT, Kontich, Belgium). Morphometric analysis was performed using CTAn software (Bruker microCT, Kontich, Belgium), and by considering the whole volume within each sample, the ratio of bone volume to total volume (BV/TV) was evaluated along with other micro-indices like trabecular thickness (Tb.Th), trabecular number (Tb.N), and trabecular separation (Tb.Sp).

All samples were preconditioned to 0.1 % apparent strain for ten cycles (Bowman et al. 1994) and were then allowed to recover for 30 min. Creep tests were then conducted by applying a uniaxial compressive ramp force corresponding to 0.2 % (2000 ${{\upmu }{\upvarepsilon }}$) of elastic strain at strain rate of 0.01 s$^{-1}$ using Zwick material testing machine (Zwick Roell, Herefordshire, UK). The force corresponding to 0.2 % compressive strain was held constant for 200 s before unloading to zero. Preliminary tests showed that the creep rate becomes constant in less than a minute. Therefore, the creep strain response was measured during the creep load for 200 s. All the tests were performed in compression at ambient temperature.

### Linear viscoelastic model

At low strain levels, a number of studies have reported the stress–strain behaviour of trabecular bone to be linear (Keaveny et al. 1994; Moore and Gibson 2002). Linear time-independent elasticity has been the most common model used for trabecular bone though some studies suggest that some nonlinearity in trabecular bone is initiated even at small strain levels (Morgan et al. 2001). In this study we assumed that the trabecular bone behaves in a linear viscoelastic manner at low stress levels corresponding to physiological strain levels of 2000 ${\upmu }{\upvarepsilon }$. The uniaxial strain at time *t*, $\varepsilon (t)$, for a linear viscoelastic material, represented by a Boltzmann superposition principle, is given by (Park and Schapery 1999)

1 $$\begin{aligned} \varepsilon (t)=\int _0^t{D(t-\tau )\frac{\hbox {d}\sigma (\tau )}{\hbox {d}\tau }\hbox {d}\tau } \end{aligned}$$

where *D*(*t*) is creep compliance and $\sigma$ is the applied stress. The creep compliance *D*(*t*) can be defined by using the generalized Kelvin–Voigt model, also referred to as the Prony series, as

2 $$\begin{aligned} D(t)=D_\mathrm{g} + \sum _{j=1}^{n_{\mathrm{pr}}}{D_j\left( 1-\exp (-t/\tau _j)\right) } \end{aligned}$$

where $D_\mathrm{g}$ is glassy or instantaneous elastic compliance, $D_j$ are transient retardation strengths, $\tau _j$ are retardation times, and $n_{\mathrm{pr}}$ is number of terms in the Prony series. The model parameters $D_\mathrm{g}$, $D_j$ and $\tau _j\, (j=1,2,\ldots ,n_{\mathrm{pr}})$ were determined by minimizing the error between measurements and Eq. 2 for each sample. This was achieved by using nonlinear least-squares fit method in MATLAB (MATLAB 2015) which iteratively improves the unknown parameter values by minimizing the sum of the squares of the residuals between the experimental observations and the model. The number of Prony terms, $n_{\mathrm{pr}}\,{=}\,3$, was found to be sufficient to accurately represent the experimental viscoelastic strain response for all the samples.

### Numerical interconversion

Many readers prefer to use viscoelastic properties in other formats: relaxation functions and complex material functions. For their convenience and use, creep compliance functions obtained from experimental tests were converted to other formats using methods proposed by Park and Schapery (1999). The Prony series representation of the relaxation modulus function, *E*(*t*), is given by

3 $$\begin{aligned} E(t)=E_\mathrm{e} + \sum _{i=1}^{n_{\mathrm{pr}}}{E_i\exp (-t/\rho _i)} \end{aligned}$$

where $E_\mathrm{e}$ is the equilibrium modulus, and $E_i$ and $\rho _i \,(i=1,2,\ldots , n_{\mathrm{pr}})$ are the relaxation strengths and relaxation times, respectively. Integral relationship between creep compliance *D*(*t*) and relaxation modulus *E*(*t*) based on Eq. 1 is given by

4 $$\begin{aligned} \int _{0}^{t}{D(t-\tau )\frac{E(\tau )}{\hbox {d}\tau }\hbox {d}\tau }=1 \; \;(t>0) \end{aligned}$$

The unknown set of constants $E_\mathrm{e}$, $E_i$ and $\rho _i$ ($i=1,2,\ldots , n_{\mathrm{pr}}$) in relaxation modulus function *E*(*t*) can be determined by solving the following system of equations (Park and Schapery 1999):

5 $$\begin{aligned} A_{ki}E_i=B_k \text { (summed on { i}; i=1,2,3)} \end{aligned}$$

where *k* the number of discrete sampling points, and

6 $$\begin{aligned} A_{ki}= & {} {\left\{ \begin{array}{ll} D_{\mathrm{g}}\exp {(-t_{k}/\rho _{i})}+\sum _{j=1}^{n_{\mathrm{pr}}}\frac{\rho _{i}D_{j}}{\rho _{i}-\tau _{j}}&{}\\ \quad \left( \exp {-(t_{k}/\rho _{i})}-\exp {-(t_{k}/\tau _{j})}\right) &{} \rho _{i}\ne \tau _{j}\\ D_{\mathrm{g}}\exp {(-t_{k}/\rho _{i})}+\sum _{j=1}^{n_{\mathrm{pr}}}\frac{t_{k}D_{j}}{\tau _{j}}&{}\\ \quad \left( \exp {-(t_{k}/\rho _{i})}\right) &{} \rho _{i}=\tau _{j} \end{array}\right. }\end{aligned}$$

7 $$\begin{aligned} B_{k}= & {} 1- E_\mathrm{e} \left( D_{\mathrm{g}}+\sum _{j=1}^{n_{\mathrm{pr}}}D_{j}\left( 1-\exp (-t_{k}/\tau _{j})\right) \right) \end{aligned}$$

8 $$\begin{aligned} E_\mathrm{e}= & {} \frac{1}{D_{\mathrm{g}}+\sum _{j=1}^{n_{\mathrm{pr}}}{D_j}} \end{aligned}$$

where $t_k$ denotes time points. The parameters $D_\mathrm{g}$, $D_j$ and $\tau _j$ ($j=1,2,3$) were determined from creep experiments for each sample. The sampling points were selected at $t_k=1/\omega _k=10^{k-5}$ $(k=1,\ldots ,10)$, and the relaxation time constants $\rho _i$ were determined by a root-finding method proposed by Park and Schapery (1999). The unknown set of constants $E_i$ ($i=1,2,3$) were evaluated by solving Eq. 5 using the least-squares method.

The creep compliance functions were also converted to complex material functions using (Park and Schapery 1999):

9 $$\begin{aligned}&D^{\prime }(\omega )=D_\mathrm{g}+\sum _{j=1}^{n_{\mathrm{pr}}}{\frac{D_j}{\omega ^2\tau _j^2+1}} \end{aligned}$$

10 $$\begin{aligned}&D^{\prime \prime }(\omega )=\sum _{j=1}^{n_{\mathrm{pr}}}{\frac{\omega \tau _j D_j}{\omega ^2\tau _j^2+1}} \end{aligned}$$

where $D^{\prime }(\omega )$, $D^{\prime \prime }(\omega )$ and $\omega$ are storage compliance, loss compliance and frequency, respectively. Dynamic loss tangent ($\tan \delta$), a measure of magnitude of viscoelastic effects, is the ratio of loss compliance to storage compliance as

11 $$\begin{aligned} \tan \delta =\frac{D^{\prime \prime }(\omega )}{D^{\prime }(\omega )} \end{aligned}$$

Time-independent elastic materials show zero loss tangent ($\tan \delta$) where as viscoelastic materials exhibit high values of $\tan \delta$. For example, the value for bone has been reported to be in the range of 0.01–0.04 (Lakes et al. 1979; Yamashita et al. 2001).

**Table 1:** The values of linear viscoelastic properties and microstructural indices of bovine trabecular bone

$\sigma$	$D_{\mathrm{g}} \times 10^{-4}$	$D_{j}\times 10^{-4}$			$\tau _{j}$			$E_{\mathrm{e}}$	$E_{i}$			$\rho _{i}$			$\tan \delta$	BV/TV	Tb.Th	Tb.N	Tb.Sp
		$$D_{1}$$	$$D_{2} $$	$$D_{3}$$	$$\tau _{1}$$	$$\tau _{2}$$	$$\tau _{3}$$		$$E_{1}$$	$$E_{2}$$	$$E_{3}$$	$$\rho _{1}$$	$$\rho _{2}$$	$$\rho _{3}$$					
0.64	34.36	1.84	1.25	2.48	1.54	24.42	284.12	250.43	14.94	9.15	16.53	1.46	23.59	266.41	0.026	0.19	0.17	1.10	0.62
0.60	34.38	3.17	1.64	3.40	0.42	8.95	161.57	234.83	24.64	11.16	20.24	0.39	8.57	148.71	0.036	0.21	0.18	1.20	0.61
0.64	35.24	1.30	1.31	2.63	1.76	13.21	155.22	247.06	10.20	9.49	17.04	1.69	12.74	145.13	0.019	0.25	0.19	1.31	0.55
0.80	26.80	1.65	1.33	1.75	0.95	8.74	128.76	317.18	21.83	15.54	18.51	0.90	8.35	121.60	0.036	0.26	0.18	1.46	0.58
1.19	17.54	1.10	0.67	0.75	0.98	8.26	101.45	498.53	34.09	18.24	19.13	0.92	7.97	97.67	0.035	0.33	0.21	1.62	0.54
1.31	15.99	0.83	0.65	1.14	1.41	10.59	130.74	537.18	31.30	21.99	34.79	1.34	10.20	122.73	0.028	0.35	0.19	1.87	0.48
0.94	21.70	1.38	1.10	1.41	0.75	8.31	157.54	390.70	27.90	19.49	22.71	0.71	7.93	148.85	0.036	0.35	0.20	1.81	0.48
1.33	15.36	1.06	0.84	1.07	0.79	8.15	157.22	545.65	42.47	29.54	33.56	0.74	7.75	148.03	0.039	0.39	0.19	2.08	0.43
0.77	26.93	1.26	0.92	1.02	0.87	6.39	103.02	332.01	16.73	11.08	11.58	0.83	6.19	99.53	0.028	0.40	0.20	2.02	0.42
1.37	14.76	1.06	0.79	0.87	0.35	5.68	115.70	572.05	45.69	30.06	29.85	0.33	5.41	109.94	0.030	0.42	0.21	1.99	0.41
1.08	19.43	1.19	0.93	1.75	1.28	10.45	135.57	429.24	30.05	20.79	34.48	1.21	10.00	125.41	0.034	0.43	0.20	2.11	0.39
2.13	9.40	0.64	0.37	0.65	1.06	9.47	148.63	904.68	68.15	34.72	55.87	0.99	9.13	139.94	0.037	0.43	0.23	1.91	0.42
1.75	11.59	0.89	0.42	0.58	1.50	14.46	156.06	741.75	61.76	25.77	33.23	1.39	13.99	149.33	0.037	0.46	0.22	2.11	0.38

$\sigma$ [MPa] is the applied constant stress. $D_\mathrm{g}$ [1/MPa], $D_j$ [1/MPa] and $\tau _j$ [s] ($j = 1, 2, 3$) are the Prony parameters in Eq. 2, and $E_\mathrm{e}$ [MPa], $E_i$ [MPa] and $\rho _i$ [s] ($i = 1, 2, 3$) are parameters in Eq. 3. $\tan \delta$ is the loss tangent at 1 Hz, Eq. 11, and BV/TV is the bone volume fraction, Tb.Th is trabecular thickness in mm, Tb.N is trabecular number in 1/mm, Tb.Sp is trabecular separation in mm

## Results

**Fig. 1:**
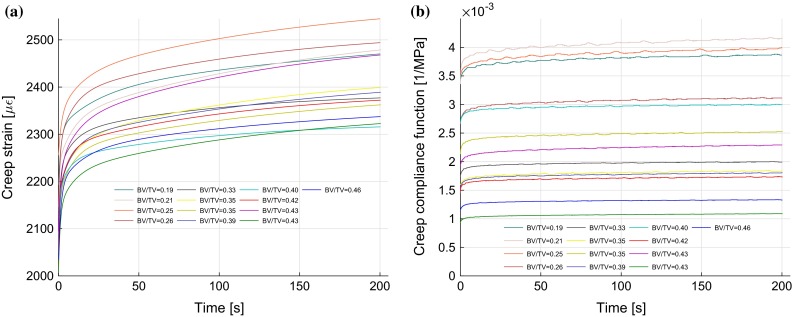
Experimental creep responses: **a** creep strain, **b** creep compliance (creep strain/applied stress) curves for all samples

The experimental creep curves corresponding to elastic strains of 2000 ${\upmu }{\upvarepsilon }$ for all samples are shown in Fig. 1a, and the corresponding compliance functions (the ratio of creep strain response to the applied stress) are plotted in Fig. 1b. Distinct creep response was clearly observed in all the samples. The BV/TV was in the range of 0.19–0.46, and their creep compliance after 200 s of constant load was in the range of $1.08\times 10^{-3}$–$4.17\times 10^{-3}\,{\hbox { MPa}}^{-1}$. Steady-state creep rate, the slope of creep strain–time curve when slope approaches to a constant in secondary creep regime, was in the range of 0.13– 0.53 ${\upmu }{\upvarepsilon }{/}$s.

### Viscoelastic compliance function

**Fig. 2:**
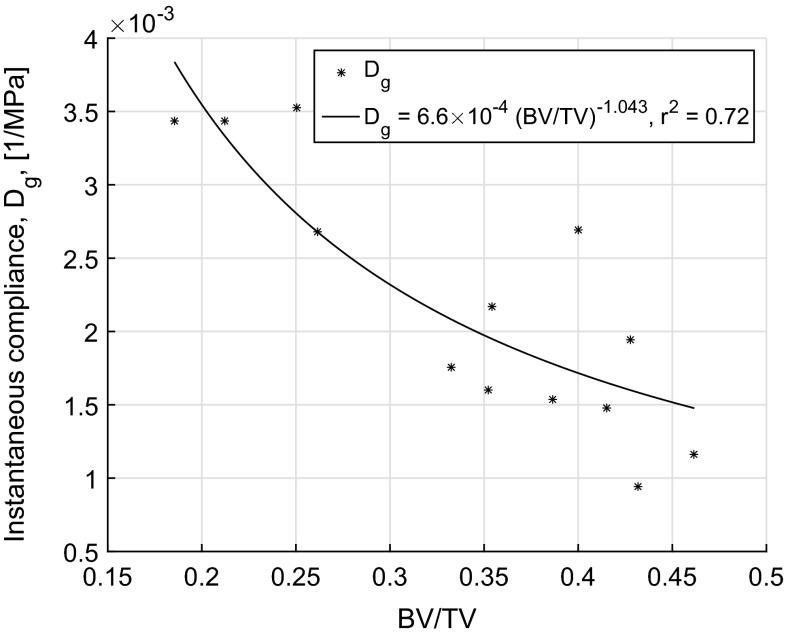
Instantaneous compliance, $D_\mathrm{g}$, plotted against BV/TV with power law relationship, $D_\mathrm{g}=6.6\times 10^{-4}(\hbox {BV}/\hbox {TV})^{-1.043} (r^2=0.72, p<0.001$)

The linear viscoelastic model parameters based on 3-term Kelvin–Voigt model or Prony series were evaluated by minimizing the error between measurements, Fig. 1b, and Eq. 2 for each sample and are presented in Table 1. The glassy or instantaneous compliance ($D_\mathrm{g}$) was in the range of $9.40\times 10^{-4}$–$34.36\times 10^{-4} \,{\hbox {MPa}}^{-1}$ and was found to decrease with increasing BV/TV ($\phi$) with a power law relationship, $D_\mathrm{g}=6.6\times 10^{-4}(\phi )^{-1.043}$ ($r^2=0.72$, $p<0.001$) as shown in Fig. 2. This relationship is similar to the previously reported modulus–density relationships in the literature for trabecular bone (Currey 1986; Keller 1994; Morgan et al. 2003). Similarly, the relationships of Tb.Th, Tb.N and Tb.Sp with $D_\mathrm{g}$ were found to be $D_\mathrm{g}=6.15\times 10^{-6}(\text {Tb.Th})^{-3.568} \ (r^2=0.57,\ p<0.001), D_\mathrm{g}=4.27\times 10^{-3}(\text {Tb.N})^{-1.358}\ (r^2=0.69,\ p<0.001$) and $D_\mathrm{g}=8.14\times 10^{-3}(\text {Tb.Sp})^{1.854}\ (r^2=0.60, \ p<0.001$), respectively. The BV/TV, among all the evaluated micro-indices, was found to be a better predictor of $D_\mathrm{g}$ with $r^2$ value of 0.72. We also examined the predictive power of BV/TV by including these indices in a multi-variable power law relationships and found no improvement. Consequently, we considered the relationship between the time-dependent behaviour and BV/TV, assuming that the latter was the lone predictor of viscoelastic response. By minimizing the error using nonlinear least squares, the relationship between creep compliance function, *D*(*t*), and BV/TV was found to be ($r^2=0.73$, $p<0.001$)

12 $$\begin{aligned} D(t)=A\phi ^m+A\left[ \sum _{j=1}^{3}{\tilde{D}_{j}\left( 1-\exp (-t/\tilde{\tau }_j)\right) \phi ^{m_t}}\right] \end{aligned}$$

**Table 2:** Power law relationship parameters: ${\tilde{D}_j}$ and ${\tilde{\tau }_j}$ ($j = 1, 2, 3$) are dimensionless transient compliance and retardation time coefficients in sec, respectively

Function	Equation	Parameters		
Creep compliance function *D*(*t*)	Equation 12	$A=6.6\times 10^{-4}$	$m=-1.033$	$m_t=-1.058$
		${\tilde{D}_1}= 0.026$	${\tilde{D}_2}=0.071$	${\tilde{D}_3}=0.093$
		${\tilde{\tau }_1}=14.237$	${\tilde{\tau }_2}=1.255$	${\tilde{\tau }_3}=250.0$
Relaxation modulus function *E*(*t*)	Equation 13	$$B=2043.0$$	$$p=1.414 $$	$$p_t=1.014$$
		${\tilde{E}_1}= 0.028$	${\tilde{E}_2}=0.049$	${\tilde{E}_3}=0.039$
		${\tilde{\rho }_1}=8.828$	${\tilde{\rho }_2}=0.929$	${\tilde{\rho }_3}=133.23$

$${\tilde{E}_i}$$ and $${\tilde{\rho }_i}$$ ($$i = 1, 2, 3 $$) are dimensionless transient relaxation moduli and relaxation time constants in sec, respectively. *A* and *B* are constants in 1/MPa and MPa, respectively. *m*, $$m_t$$, *p* and $$p_t$$ are dimensionless power law coefficients

where $\tilde{D_j}$ ($j = 1, 2, 3$) represent the dimensionless transient compliance coefficients expressed as fractions of instantaneous compliance, ${\tilde{\tau }}_j$ (*j* = 1, 2, 3) are time coefficients and *A*, *m*, and $m_t$ are constants. All the evaluated parameters are reported in Table 2. Three samples with BV/TV of 0.26, 0.35 and 0.46, one sample from each of the femoral head and one sample from a trochanter, were chosen to show the representative behaviour of the samples. The predicted viscoelastic response is shown in Fig. 3 for these three samples. The maximum errors between the measured and the predicted values from Eq. 12 were $-1.8, -11.6\ \hbox {and}\ 28.8\,\%$ with BV/TV of 0.26, 0.35 and 0.46, respectively. The negative error value indicates the under-prediction of the power law model, whereas positive error indicates the over-prediction of the model compared to the experimentally measured viscoelastic response. The overall coefficient of determination ($r^2$) for the pooled data comprising 13 samples was 0.73.

**Fig. 3:**
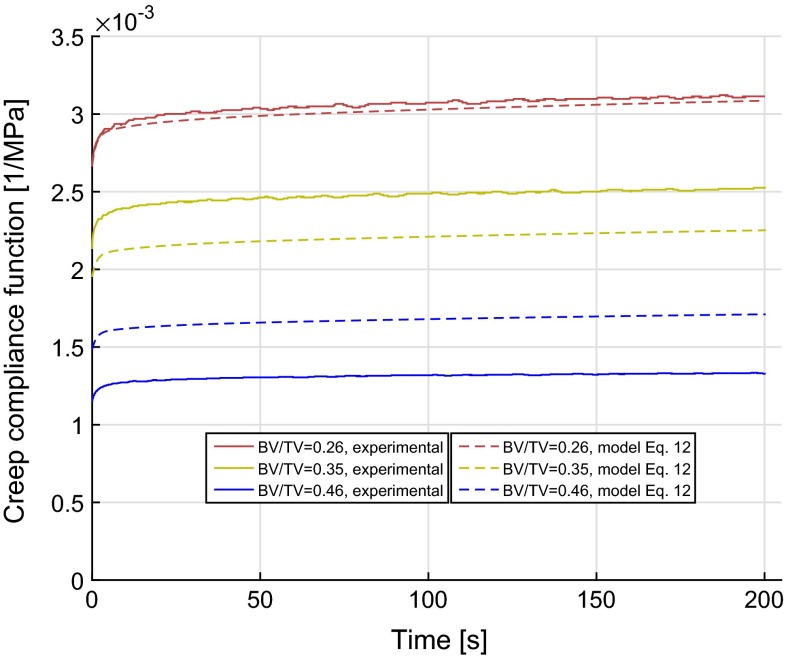
Time-dependent creep compliance function, *D*(*t*), with time for three samples. *Dotted lines with same colour* show the predictions from regression model, Eq. 12. The coefficient of determination $r^2$ was 0.73 ($p<0.001$).

**Fig. 4:**
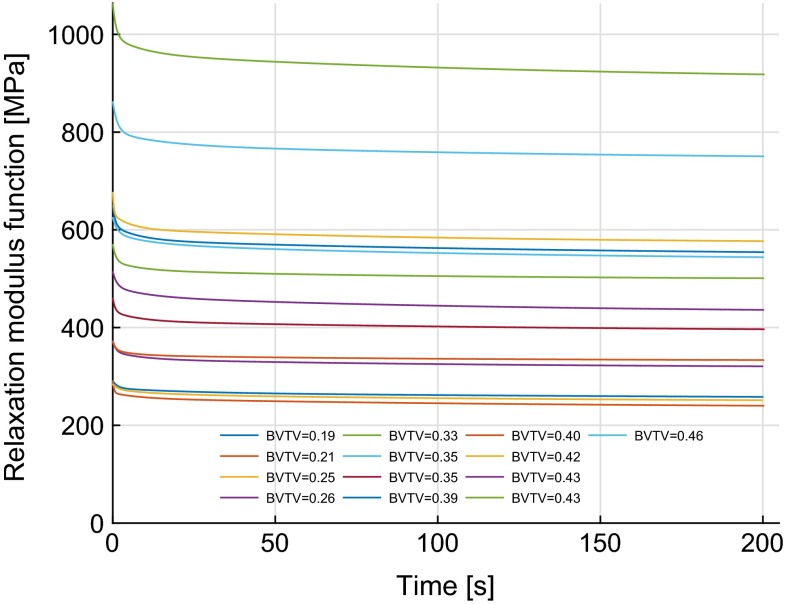
Time-dependent relaxation function with time for all samples

### Viscoelastic modulus function

**Fig. 5:**
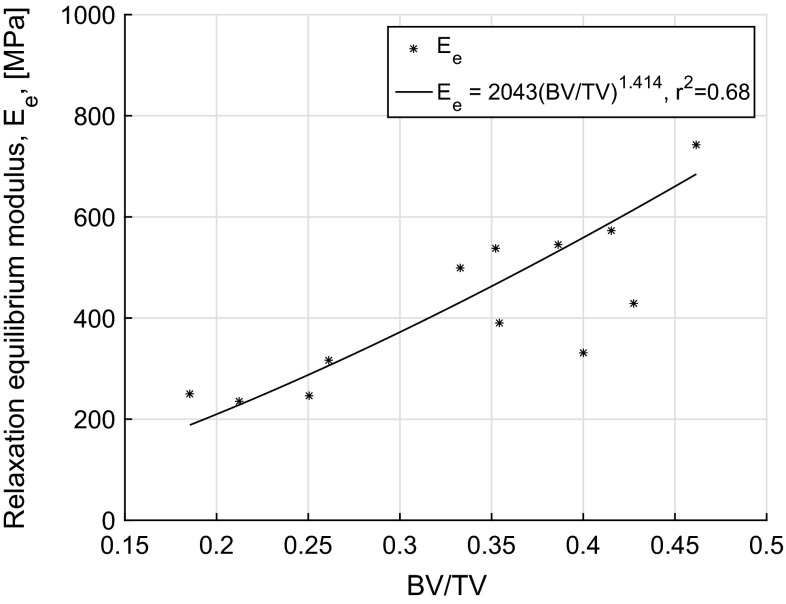
Equilibrium relaxation modulus, $E_\mathrm{e}$, plotted against BV/TV with power law relationship, $E_\mathrm{e}=2043(\hbox {BV}/\hbox {TV})^{1.414}$ ($r^2=0.68$, $p<0.001$)

The creep compliance functions were converted to time-dependent relaxation functions using numerical interconversion methods as discussed and the resulting relaxation modulus functions are shown in Fig. 4. The long-term or equilibrium modulus ($E_\mathrm{e}$) for all samples was in the range of 234.8–904.6 MPa and was found to follow a power law relation with BV/TV as shown in Fig. 5. Using an approach similar to that used for compliance functions, a relationship between time-dependent relaxation modulus function, *E*(*t*), and BV/TV, $\phi$, over time was found ($r^2=0.68$, $p<0.001$).

13 $$\begin{aligned} E(t)=B\phi ^p+B\left[ \sum _{i=1}^{3}{\tilde{E}_{i}\exp (-t/\tilde{\rho }_i)\phi ^{p_t}}\right] \end{aligned}$$

**Fig. 6:**
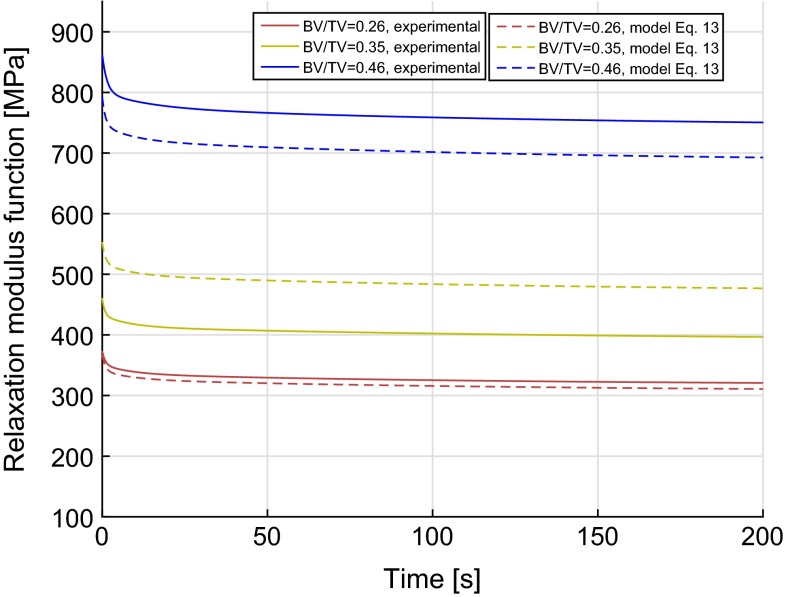
Time-dependent relaxation function, *E*(*t*), with time for three samples. *Dotted lines with same colour* show the predictions from regression model from Eq. 13. The coefficient of determination $r^2$ was 0.68 ($p<0.001$).

where $\tilde{E_i}$ represents the dimensionless transient moduli and are expressed as fractions of equilibrium modulus, $\tilde{\rho _i}$ (*i* = 1, 2, 3) are time coefficients, and *B*, *p* and $p_t$ are constants. All evaluated parameters are reported in Table 2, and the resulting predicted viscoelastic response is shown in Fig. 6 for samples with BV/TV of 0.26, 0.35 and 0.46. For these three samples, the maximum errors between the measured and the predicted values from Eq. 13 were 3.1, $-20.3$, and 8.4 % with BV/TV of 0.26, 0.35, and 0.46, respectively. The coefficient of determination ($r^2$) for the pooled data comprising 13 samples was 0.68. The above can also be represented using a rheological model as shown in Fig. 7.

**Fig. 7:**
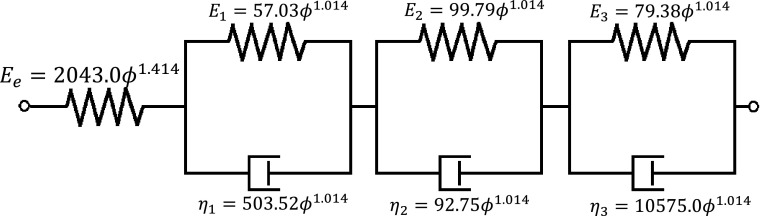
Kelvin–Voigt rheological model and the relationships of its associated parameters with BV/TV. $E_\mathrm{e}$, $E_1$, $E_2$ and $E_3$ represent elastic moduli in MPa. $\eta _1$, $\eta _2$ and $\eta _3$ represent viscosity coefficients in MPa.s.

### Loss tangent

**Fig. 8:**
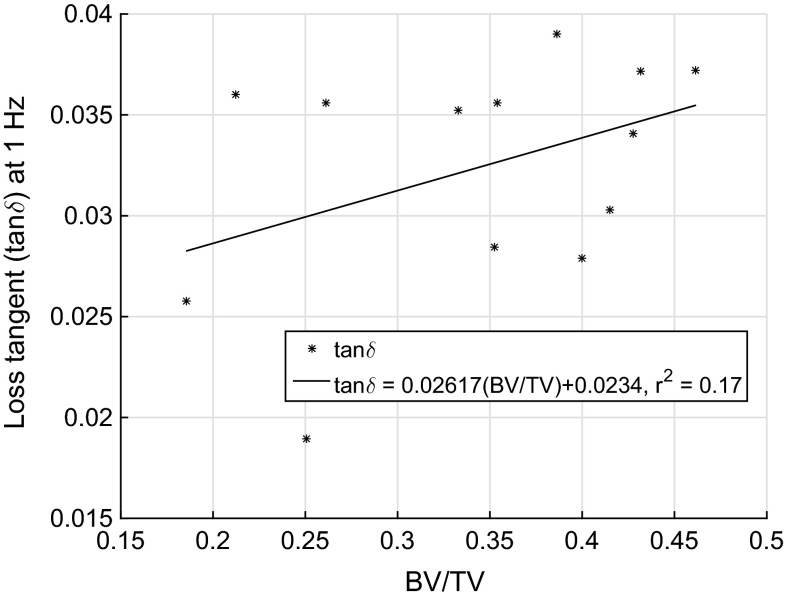
Loss tangent at driving frequency of 1 Hz. There was no significant relationship found between the loss tangent and the BV/TV, $\tan \delta = 0.02617(\hbox {BV}/\hbox {TV})+0.0234$ ($r^2=0.17$, $p=0.16$).

The creep compliance of each sample was converted to complex storage modulus, loss modulus and loss tangent at a driving frequency of 1 Hz using Eqs. 9, 10 and 11, respectively. The loss tangent was found to be between 0.019 and 0.039 for all samples, similar to the values reported previously by Guedes et al. (2006). There was no statistically significant relationship ($r^2=0.17$, $p=0.16$) found between the loss tangent and the BV/TV (Fig. 8).

## Discussion

The trabecular bone has been investigated extensively for its mechanical properties, but its time-dependent behaviour has received relatively little attention (Deligianni et al. 1994; Bowman et al. 1994; Yamamoto et al. 2006; Quaglini et al. 2009). The relationships between time-independent elastic modulus and BV/TV (or density) have been reported extensively over the last two decades (Currey 1986; Keller 1994), but similar relationships of BV/TV with viscoelastic properties have not been previously investigated to the best of our knowledge.

In this study, we conducted creep experiments on bovine trabecular bone samples, and the measured behaviour was quantified using linear viscoelastic theory based on 3-term Prony model (generalized Kelvin–Voigt model). Our study shows that bone volume fraction can be significantly related to creep compliance and relaxation modulus functions with the coefficients of determination of 0.73 and 0.68 ($p<0.001$), respectively. It is important to note that similar $r^2$ values have been previously reported by studies that relate bone density to time-independent elastic modulus (Currey 1986; Morgan et al. 2003). In fact the instantaneous elastic compliance-BVTV relationship from the creep experiments conducted in this study is similar to the relations reported in the literature (Zysset 2003) with similar $r^2$ value.

The creep strain response was to found to reach the secondary creep regime with the steady-state creep rate (constant slope) in under 1  min for all samples and the chosen 200 s duration was, therefore, sufficient for the determination of linear viscoelastic properties. Bowman et al. (1994) experimentally observed the creep behaviour of trabecular bone until failure at different applied normalized stress levels (0.5–1% elastic strains) and concluded that the creep behaviour of trabecular bone is nonlinearly dependent on applied stress level. Since our tests were performed at relatively low stresses (maximum creep strain was under 0.26 %, Fig. 1a), and we believe that the assumption of linear viscoelasticity is valid.

The popular FE-based simulations mostly consider the trabecular bone as elastic material (Pankaj 2013), but to predict the stability of joint replacement and fracture fixation implants, it is necessary to consider viscoelastic properties for trabecular bone in FE simulations. Most commercial FE packages have viscoelastic modelling capabilities. With existing imaging capabilities, subject-specific BV/TV values can be estimated permitting the application of viscoelastic properties based on the relationships developed in this study in finite element models if time-dependent behaviour of trabecular bone is of concern. Trabecular bone provides anchorage to orthopaedic implants, whose stability depends not only on bone quality but also on its relaxation or creep behaviour (Norman et al. 2006). It has been proposed that the age related nontraumatic fractures in vertebra and height loss are related to long term accumulated creep strains (Pollintine et al. 2009; Luo et al. 2012). The major role of trabecular bone is not only transferring the load but to dissipate energy during daily activities thereby protecting the articular cartilage as well at the ends of long bones (Linde et al. 1989). So the models developed in this study are likely to be used in FE simulations aimed at enhancing the understanding of the above and other clinical problems involving trabecular bone. It is important to note that the relationships found here were developed using specimens that may be much larger than the typical element size employed in whole-bone FE analyses. However, what we are proposing is similar to the current practice of assigning time-independent elastic moduli in macro-mechanical FE models. The current practice is to use elastic properties based on density-modulus relationships that are almost always established using experiments or simulations on larger samples (Morgan et al. 2003). Even when macro-mechanical properties are determined from micro-FE analyses (models developed from micro-CT scans), typical volume sizes need to be around 5 mm or more (Harrigan et al. 1988).

It is recognized that the individual constituents at different hierarchical levels in the trabecular bone and its microstructure contribute to the overall viscoelastic behaviour at the specimen level. The contribution of these constituents to the viscoelastic behaviour is beyond the scope of this paper. However, from our results, the microarchitectural indices (Tb.Th, Tb.N, Tb.Sp and BV/TV) significantly relate to mechanical behaviour of the trabecular bone, and it is evident that among all the micro-indices the BV/TV plays a major role in predicting the viscoelastic behaviour. The lower bound of the time range of the retardation spectrum $\tau _1$, in Table 1, was in the order of 0.35 s while $\tau _2$ and $\tau _3$ were in the order of 5.6 and 101.4 s, respectively. Since $\tau _1$ is quite small even relatively fast strain rates ($0.01\,\hbox {s}^{-1}$, i.e., 0.2 s to reach $2000\,{\upmu }{\upvarepsilon }$ in our tests) may allow some creep during the finite ramp loading. Further tests at higher and lower strain rates are necessary to verify this. The small values of $\tau _1$ indicate that the trabecular bone experiences some part of its creep deformation or stress relaxation in a relatively short time period.

Our study also has a few limitations. Firstly, we have performed creep tests on bovine samples as they were readily available. Morgan et al. (2003) reported that the time-independent modulus–density relationships depend on anatomic site. Whether the viscoelastic-BV/TV relationships depend on anatomic site and/or species is a topic of future research. Secondly, as in many previous studies, our experiments were performed at room temperature. It is possible that increase in temperature to $37\,^{\circ }\mathrm{C}$ may have a small effect on the creep behaviour; currently the published data to confirm or invalidate this is limited.

## Conclusions

We have performed uniaxial creep experiments on cylindrical bovine trabecular bone samples to quantify the viscoelastic properties. These properties significantly relate to the BV/TV with power law relationships ($r^2=0.73, p<0.001$) and can be used readily in finite element simulations involving trabecular bone.
